# Epidemic Dysentery

**Published:** 1873-11

**Authors:** J. M. Hall


*MEDICAL & SURGICAL REPORTER:*

Epidemic Dysentery.—*J. M. Hall, M.D.*—The months of August and September of this autumn will long be remembered by the people of Fayetteville, Ohio, for the general prevalence of the "Bloody Flux." There is scarcely a family for as much as from fifteen to twenty miles round our place that have not suffered more or less, in some form or other, from the affection. The remedies for treatment relied on by us were castor oil sufficiently often to keep the bowels free, and opium or some of its preparations, combined with tannin and bismuth, were used to the exclusion of almost any other remedy. Those cases troubled with vomiting were very soon and effectually relieved by the application of mustard to the stomach and the administration of small and repeated doses of pepsin and bismuth. The foregoing medication, coupled with perfect rest, abstinence from liquids, a light but nourishing diet, formed the routine of treatment, from which satisfactory results were obtained.

